# Tracing and regulating redox homeostasis of model benthic ecosystems for sustainable aquaculture in coastal environments

**DOI:** 10.3389/fmicb.2022.907703

**Published:** 2022-08-10

**Authors:** Nobuaki Shono, Mana Ito, Akio Umezawa, Kenji Sakata, Ailong Li, Jun Kikuchi, Katsutoshi Ito, Ryuhei Nakamura

**Affiliations:** ^1^Biofunctional Catalyst Research Team, Center for Sustainable Resource Science, RIKEN, Wako, Japan; ^2^Fisheries Technology Institute, Japan Fisheries Research and Education Agency, Hatsukaichi, Japan; ^3^RIKEN Center for Sustainable Resource Science, Yokohama, Japan; ^4^Graduate School of Medical Life Science, Yokohama City University, Yokohama, Japan; ^5^Graduate School of Bioagricultural Sciences, Nagoya University, Nagoya, Japan; ^6^Earth-Life Science Institute, Tokyo Institute of Technology, Tokyo, Japan

**Keywords:** redox homeostasis, benthic ecosystems, aquaculture, electromicrobiology, benthos

## Abstract

Aquaculture in coastal environments has an increasingly important role in the world’s food supply; however, the accumulation of organic compounds on seafloors due to overfeeding adversely affects benthic ecosystems. To assess the ecological resilience of aquafarms to nutrient influx, we investigated the redox homeostasis of benthic ecosystems using a marine oligochaete as a model benthic organism in aquaculture fields. Real-time monitoring of the redox potential of a model benthic ecosystem constructed in an electrochemical reactor allowed evaluation of the homeostatic response of the system to nutrient addition. Although the detrimental effects of overfeeding were confirmed by irreversible potential changes in the sediment, redox homeostasis was reinforced through a cooperative relationship between oligochaetes and sediment microorganisms. Specifically, the oligochaetes exhibited reversible changes in metabolism and body position in response to dynamic changes in the sediment potential between −300 and 500 mV, thereby promoting the decomposition of organic compounds. The potential-dependent changes in metabolism and body position were reproduced by artificially manipulating the sediment potential in electrochemical reactors. Given the importance of benthic animals in sustaining coastal ecosystems, the electrochemical monitoring and physiologic regulation of marine oligochaetes could offer an intriguing approach toward sustainable aquaculture.

## Introduction

The United Nations estimates that the world population will reach 9.8 billion in 2050 and result in an unprecedented food crisis (United Nations, Department of Economic and Social Affairs, Population Division, 2015). To increase food stability, the sustainable use of marine resources, mainly through aquaculture, is considered to be essential (Costello et al., 2020). Currently, half of the fish and shellfish consumed in the world are sourced from aquafarms, which have production rates that are growing faster than those in any other animal production sector (FAO, 2014). In addition, it has been estimated that approximately 15 billion tons of fish can be produced annually in marine coastal areas through aquaculture (Gentry et al., 2017). However, the feeding practices for aquaculture fields located in marine coastal areas often result in overfeeding and the accumulation of organic matter in seafloor sediments, which adversely affects benthic ecosystems (Yokoyama et al., 2006; Holmer et al., 2008a) and leads to eutrophication, algal blooms, and red tides (Sarà, 2007). A recent evaluation of the aquaculture industry using a composite sustainability index comprised of food, energy, water, and carbon, revealed that most aquaculture practices worldwide has low sustainability indices (Jiang et al., 2022). Therefore, there is a need to establish sustainable aquaculture systems that balance productivity and environmental conservation.

Traditionally, the environmental assessment of aquaculture fields uses both chemical indicators and benthic fauna (Holmer et al., 2008b), with the latter serving as bioindicators for collecting diverse information of aquatic environments (Martinez-Crego et al., 2010). However, this approach is not suitable for assessing short-term environmental changes, such as those that occur on the hour- and day-scale. Although chemical indicators, such as oxygen, nitrogen, phosphorus, and sulfur compounds have the potential to instantly respond to changes caused by feeding, this method requires enormous efforts for on-site water and sediment sampling and the comprehensive analysis of complex chemical compounds.

As an alternative approach to bioindicator and chemical analyses, we applied electrochemical techniques for assessing the resilience of benthic ecosystems in aquaculture farms (Ito et al., 2019). The environmental redox potential is a physicochemical value determined by the integration of various factors, including (i) biological parameters, such as gene expression, metabolism, and bacterial flora; (ii) chemical parameters, such as oxygen, sulfide, and nutrient concentrations; and (iii) nutrient influx from rivers and land due to human activities. Therefore, real-time monitoring of the environmental redox potential can be used to evaluate the overall and homeostatic responses of benthic ecosystems associated with aquaculture to feeding.

Redox homeostasis is a common phenomenon in microorganisms and is primarily maintained at the cellular level through the regulation of metabolism and gene expression. At the ecosystem level in aquaculture fields, such homeostasis has the potential to be further strengthened through the cooperative relationship between microorganisms and benthic animals, such as shrimp, polychaetes, and bivalves (Levin et al., 2001; Marinelli and Waldbusser, 2005). As redox homeostasis is disrupted by overfeeding, understanding how the homeostasis is sustained by the interplay between benthic animals and microorganisms is fundamental for developing sustainable aquaculture systems.

As a proof of concept for the use of real-time monitoring of environmental redox potential to assess ecosystem-level homeostasis, here, we reconstructed model benthic ecosystems inside an electrochemical (EC) reactor. A chemically stable and biocompatible conductive glass was used as an electrode within marine sediment containing the marine oligochaete *Thalassodrilides* cf. *briani* (Torii et al., 2016). Aquatic oligochaetes (Annelisda, Clitellata) are found in marine, estuarine, and freshwater environments worldwide (Timm, 1980) and are recognized as ecosystem engineers owing to their bioturbation and organic decomposition activities (Jones et al., 1994; Mermillod-Blondin and Rosenberg, 2006). Using *T*. cf. *briani* as a representative benthos in aquaculture fields, we conducted real-time monitoring of redox homeostasis and analyzed how the redox potential is affected by feeding and the metabolic states and movement of oligochaetes. Utilizing electrochemical techniques, we also artificially manipulated the sediment potential to investigate if the metabolic states and macroscopic movement of oligochaetes could be regulated.

## Materials and methods

### Sediment sampling and maintenance

Sediment was obtained below a fish culturing raft in the coastal area of Ehime, Japan. The sediment sample was stored in the dark at 19°C with artificial seawater (MARINE ART SF-1; Osaka Yakken Co. Ltd., Osaka, Japan). Prior to experimental use, the sediment was transferred into a beaker at a thickness of approximately 2 cm and covered with approximately 5 cm of artificial sea water.

### Preparation of sediment samples

A portion of the sediment being maintained in artificial seawater was collected onto a plate and benthic organisms visible to the naked eye were manually removed. This sediment was considered to contain only microorganisms and a portion of this sample was autoclaved at 121°C for 20 min to prepare sediment without microorganisms or benthos. Finally, forty *T*. cf. *briani* specimens were added to both the autoclaved and microorganisms-only sediment to generate sediment samples that contain only benthos and microorganisms and benthos, respectively.

### Electrochemical experiment

A three-electrode electrochemical cell (EC; 8 ml capacity) was assembled using a platinum wire and Ag/AgCl/saturated KCl as counter and reference electrodes, respectively. A fluorine-doped tin oxide (FTO)-coated glass electrode (surface area of 3.14 cm^2^; SPD Laboratory, Inc.) was used as a working electrode and was placed at the bottom of the EC reactor. The 3 g of wet sediments and 4 ml of artificial sea water was added in the EC reactor. The temperature of the reactor was maintained at 25°C and no agitation other than that resulting from experimental operation was made during the measurements. The head space of the EC reactor is open to air *via* a membrane filter (0.20 μm pore size; ADVANTEC). Redox potential measurements were conducted using open circuit potential measurements performed with an automatic polarization system (HZ-5000; Hokuto Denko). As nutrients, fish feed (dry pellet; Otohime B1; Marubeni Nisshin Feed, Tokyo, Japan) was pulverized into a powder and suspended in artificial seawater (MARINE ART SF-1; Osaka Yakken Co. Ltd., Osaka, Japan) at a concentration of 20 mg/ml. The amount of fish food in the EC reactor was adjusted by varying the amount of suspension added, and the resulting air-saturated suspension was added to the electrochemical cell. A current vs. time curve was measured under potentiostatic conditions at 400 or 0 mV (vs. SHE) using the automatic polarization system.

### NMR analysis

The collected oligochaetes from the EC reactor were gently washed with artificial seawater to remove excess material and transferred to a 2-ml safe lock tube. As much artificial sea water was removed from the tubes as possible by aspiration, and the tubes were allowed to vacuum dry using a GLD-136C rotary vacuum pump (ULVAC), and were then stored at −80°C. Prior to use, 3 mm × 2 mm zirconia beads were added to the tubes and the dried mass of oligochaetes was crushed with a Bead Smash 12 (TOMY) at 3,000 rpm for 5 min. As a calibration standard, 0.1 mM DSS (4,4-dimethyl-4-silapentane-1-sulfonic acid) in D_2_O (pH = 7, adjusted with 100 mM potassium phosphate buffer; Yoshida et al., 2014) was added to the samples, which were then incubated at 60°C for 15 min with frequent mixing. The samples were centrifuged at 15,000 rpm for 10 min at room temperature, and the supernatant was transferred to a new tube and stored at either 4°C or frozen. Prior to analysis, the samples were centrifuged at 15,000 rpm for 10 min at room temperature and transferred to 5-mm Φ—nuclear magnetic resonance (NMR) tubes (Shigemi). All oligochaetes samples were subjected to 2D-*J*res NMR analysis for the monitoring of temporal metabolic changes following the electrochemical experiments conducted in sediment. ^1^H-^13^C-HSQC and ^1^H-^13^C-HSQC-TOCSY NMR was then applied to a standard sample (oligochaetes prior to experimental use) for the NMR signal assignment of metabolites.

### Acquisition and analysis of time lapse images

To acquire time lapse images, an electrochemical cell (1 cm × 3.14 cm; same area as used in the electrochemical experiments above) with a flat side was prepared and into which 3 g sediment, 3 ml artificial seawater, and 10 oligochaetes were added. The prepared cell was placed in a box equipped with a mild light source and a time-lapse camera (TLC 200 PRO) in the front, and images were then acquired while conducting potential and current measurements. Image analysis was performed using ImageJ software and a macro program for counting the number of oligochaete tails and quantifying sediment movement.

## Results and discussion

### Real-time monitoring of redox homeostasis

**Figure 1:**
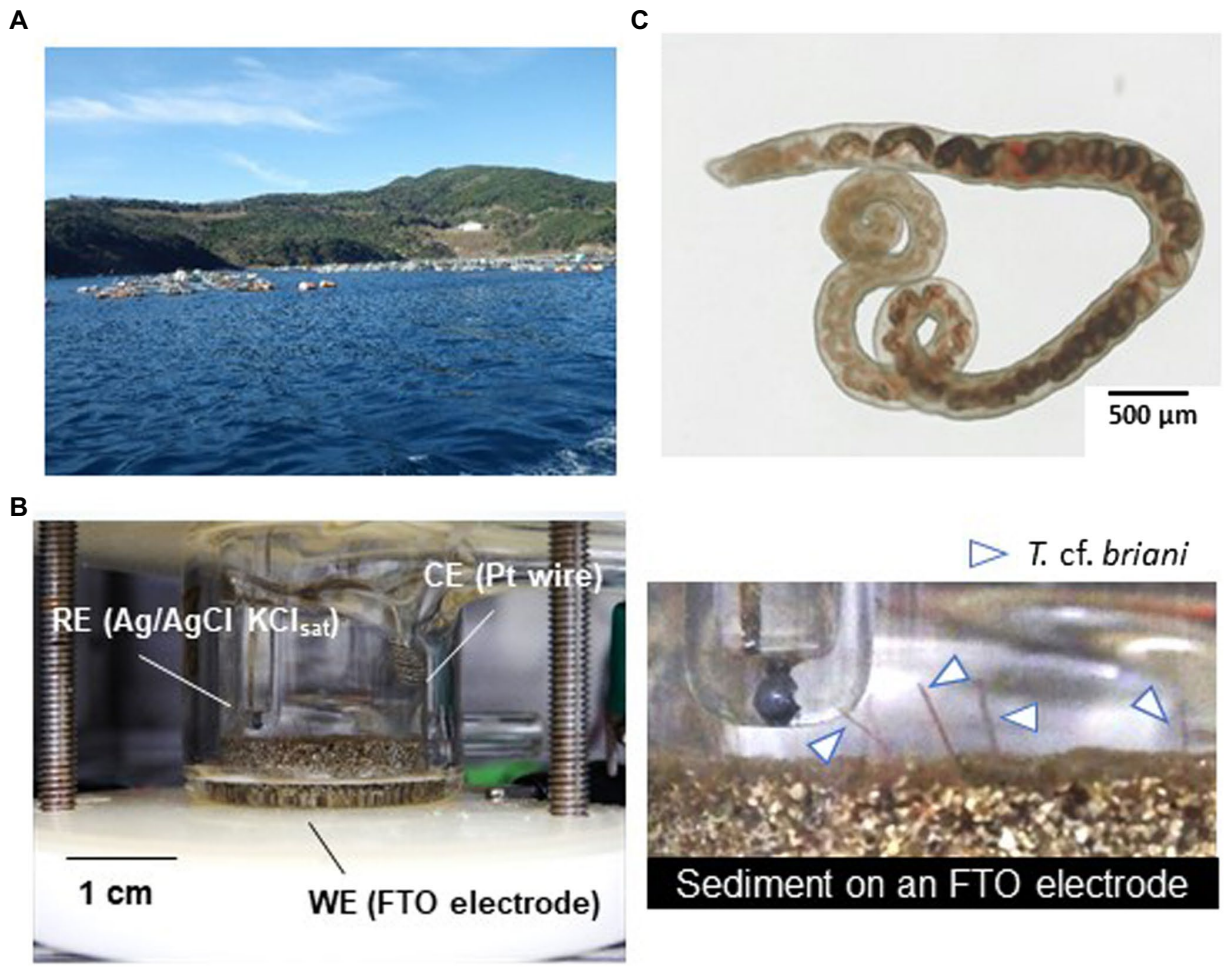
**(A)** Photograph of the fish farm where the sediments used in this study were collected. **(B)** Photographs of the model benthic ecosystem constructed in an electrochemical reactor. RE, WE, and CE denote the reference, working, and counter electrodes, respectively. The objects indicated by the arrows are the tails of oligochaetes protruding from the sediment mounted on the surface of a fluorine-doped tin oxide (FTO)-coated glass electrode. **(C)** Light microscope image of the specimen of the marine oligochaete *Thalassodrilides* cf. *briani*, which was located in sediment samples collected directly beneath the aquaculture raft.

To construct a miniature benthic ecosystem for real-time monitoring, marine sediments collected approximately 30 m directly beneath an aquaculture raft were placed in an EC reactor equipped a glass electrode on the bottom surface (Figure 1A). The glass working electrode was coated with fluorine-doped tin oxide (FTO), which has high biocompatibility and long-term chemical durability (Ishii et al., 2015), and Ag/AgCl (KCl sat.) was used as the reference electrode (Figure 1B). In this study, the term “redox potential” represents the electrochemical potential measured under open circuit conditions.

**Figure 2:**
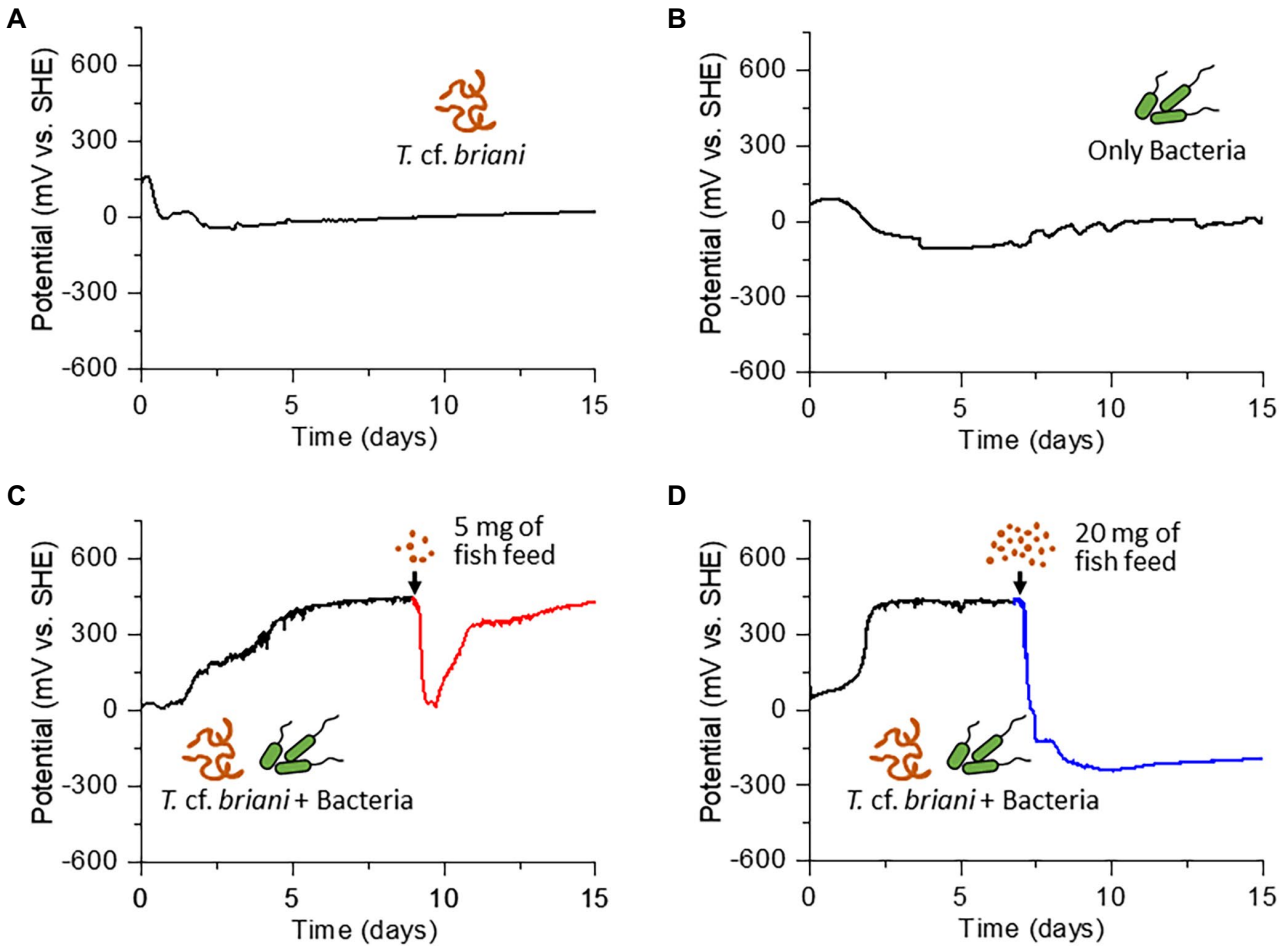
Time courses of open circuit potential measurements for **(A)** sediment containing only oligochaetes, **(B)** sediment containing only sediment microorganisms, and **(C,D)** sediment containing both oligochaetes and sediment microorganisms. The arrow in **(C)** and **(D)** indicates the time at which 5 and 20 mg of fish diet, respectively, was added to the electrochemical reactor.

The dominant benthic organism in the sediment was a marine annelid identified as *T.* cf. *briani* (Torii et al., 2016), which is a few centimeters in length and a member of the subclass Oligochaeta. *Thalassodrilides* cf. *briani* was selected as a representative benthic invertebrate for this study and other animals, such as polychaetes and amphipods, were removed from the sediment samples (Figure 1C). *Thalassodrilides* cf. *briani* is present at high densities in sediments located in the vicinity of fish farms (Ito et al., 2018), has high tolerance to concentrated sulfide species, and is able to remediate organically polluted sediments (Ito et al., 2016). Cooperative relationships between benthic animals and sediment microorganisms play a fundamental role in maintaining aquaculture environments by accelerating the decomposition of organic matter that accumulates on the seafloor (Andersen and Kristensen, 1992; Nascimento et al., 2012). To examine if this relationship could be monitored electrochemically, we prepared sediment samples containing only microorganisms, only *T.* cf. *briani*, or both *T.* cf. *briani* and microorganisms, and conducted real-time potential monitoring of the three sediments under open circuit conditions for 15 days. The sediment containing only microorganisms was prepared by removing visible benthic organisms, whereas the sediment containing only benthos was generated by adding *T.* cf. *briani* to autoclaved sediments. These two sediment samples exhibited nearly identical profiles for potential, with the potentials ranging from −100 to 100 mV vs. standard hydrogen electrode (SHE) throughout the 15-day experiment (Figures 2A,B). In contrast, the sediments containing both *T.* cf. *briani* and microorganisms showed a steep increase in potential and transient spike-like potential changes of approximately 10 mV, with the potential reaching approximately 450 mV after 10 days of incubation (Figures 2C,D). The samples lacking either *T.* cf. *briani* or sediment microorganisms did not reach potentials above 100 mV. Therefore, the steep increase in potential up to 450 mV was unique to the marine sediment containing both *T.* cf. *briani* and microorganisms.

Under homeostatic conditions, an ecosystem is expected to reversibly respond to external perturbations such as nutrient influx, which would occur in aquaculture sediments during feeding. However, once an external stress exceeds the resilience limit of the ecosystem, the system can no longer return to the original state. To test if this general homeostatic property is detectable by redox potential measurements, organic matter in the form of fish diet was added into the EC reactor with sediment containing both *T.* cf. *briani* and microorganisms. When 5 mg fish diet was added to the EC reactor, the potential dropped rapidly from 450 to 0 mV; however, the potential increased rapidly in the 24 h following the nutrient addition and then gradually returned to nearly 450 mV level after 1 week of incubation, demonstrating that this model benthic ecosystem functions homeostatically (Figure 2C). Further, when the amount of fish diet added to the EC reactor was increased 4-fold (20 mg), the potential decreased to approximately −300 mV and did not recover to positive values, even after 10 additional days of incubation (Figure 2D; Supplementary Figures 1–3). Such an irreversible change of redox potential indicates that the amount of fish diet added to the reactor exceeded the capacity of this model benthic ecosystems. Consistent with this finding, the sediment in the nutrient-rich system gradually turned black due to the formation of iron sulfides and all of the marine annelids had died under the low potential conditions. Notably, when the experiment was repeated for the sediments lacking either *T.* cf. *briani* or sediment microorganisms, the potential did not recover even after 5 mg feeding, indicating that the cooperation between oligochaetes and sediment microorganisms enhanced the resilience to nutrient influx. This finding, in turn, suggests that the real-time monitoring of redox homeostasis can provide feedback on the feeding practices of aquaculture farms to avoid waste and harm to the surrounding benthic ecosystems.

### Potential-dependent movement of benthos

**Figure 3:**
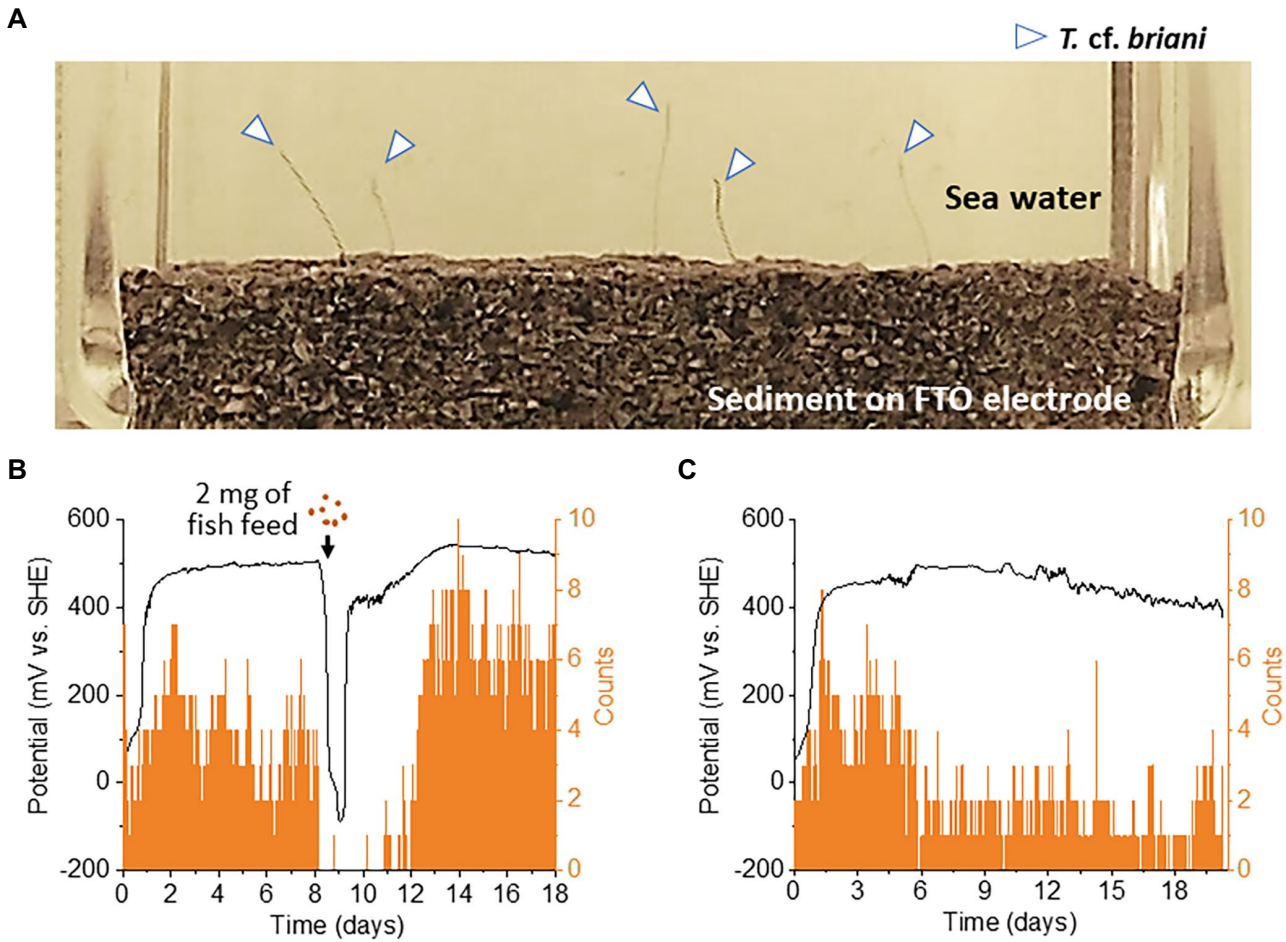
**(A)** Photograph of the seawater-sediment interface taken during open-circuit potential measurements. The arrows show the tail of an oligochaete protruding from the sediment layered on an fluorine-doped tin oxide (FTO)-coated glass electrode. **(B,C)** Correlation between redox potential and the number of tails protruding from the sediment into sea water. On day 8 of the experiment, 2 or 0 mg of fish feed (**B,C**, respectively) was added to the reactor.

Benthic animals move for a variety of reasons, including nutrient acquisition, locating suitable microhabitats, and the evasion of predators, pollutants, and adverse sediment conditions (Rodriguez and Reynoldson, 2011). As *T.* cf. *briani* is present at the seawater-sediment interface, the movement of this marine annelid is expected to have a significant influence on the redox states of benthic ecosystems. To investigate the correlation between redox potential and the movement of *T*. cf. *briani* in sediment, we developed an EC reactor equipped with an automated photography system (Figure 3A). For this experiment, 10 *T.* cf. *briani* specimens were added to the seafloor sediment on an FTO electrode, and photographs of the seawater-sediment interface were taken every 10 min while simultaneously measuring the electrochemical potential under open circuit conditions. We focused on two body positions: protrusion of the annelid tails from the sediment toward the oxygenated seawater and complete submersion in the sediment. Upon addition of *T.* cf. *briani* to the sediment, the redox potential increased from 0 V to approximately 500 mV, and this high potential value was maintained for 8 days (Figure 3B). The potential then rapidly dropped to −100 V following the addition of 2 mg fish diet, but again increased and had exceeded 400 mV within 24 h. Notably, the change in redox potential was highly synchronized with the macroscopic movement of *T.* cf. *briani*. Specifically, when the potential reached approximately 500 mV, nearly half of the oligochaetes had their tails projecting toward the seawater. Upon feeding, however, the oligochaetes began digging into the sediment until their bodies were completely buried. When the sediment potential recovered to the original state, the oligochaetes again began to stick their tails out into the seawater, demonstrating that the macroscopic movement of this benthos is highly correlated with the homeostatic changes in the redox potential. After prolonged incubation without feeding, the number of oligochaetes with protruding tails gradually decreased from 8 to 2 (Figure 3C), indicating that the “tail upwards” movement is correlated to energy metabolism. This speculation was examined below by the NMR analysis of metabolites.

The bioturbation and burrowing of aquatic oligochaetes is known to alter seawater and sediment fluxes, which could help create an oxidative environment by promoting the influx of oxygen into the sediment (Mermillod-Blondin and Rosenberg, 2006). To estimate the aeration effect caused by the macroscopic movement of *T.* cf. *briani*, the seawater-sediment interface in the EC reactor was mechanically agitated using a microsyringe at an injection rate of 0.2 ml/s, which is much more vigorous than that caused by the movement of *T.* cf. *briani*. A video showing *T.* cf. *briani* digging into the sediment is available in Supplementary Video 1. During the agitation resulting from the pipetting, a significant amount of sediment was displaced into the seawater; however, the potential only increased by approximately 70 mV, and quickly returned to its original level of approximately −20 mV within 3 min (Supplementary Figure 4). Even after multiple agitation cycles, we were unable to reproduce the sustained increase in the sediment potential above 400 mV that was observed in the reactor containing both *T.* cf. *briani* and microorganisms. Therefore, the decomposition of organic matter by digestive activity, rather than mechanical aeration effects by the movement of benthos, is the primary reason for the generation of the oxidative environment in our model benthic ecosystem.

### Redox-dependent energy metabolism

**Figure 4:**
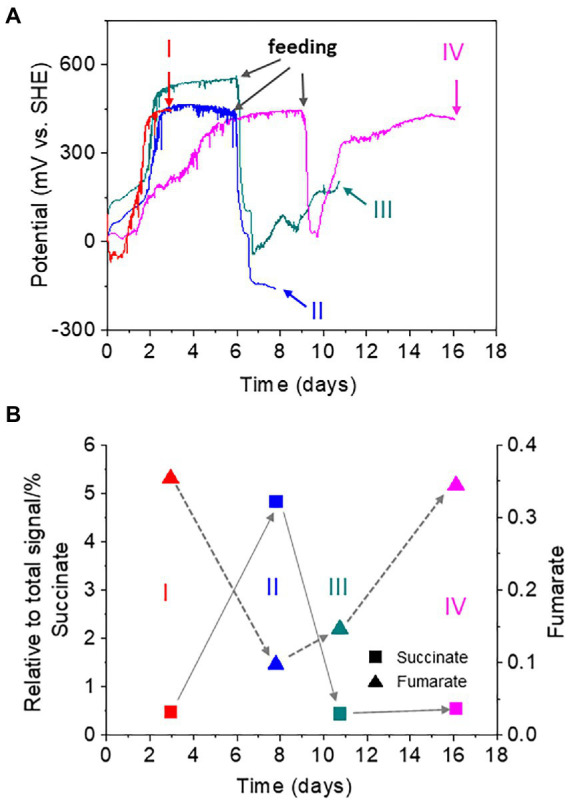
**(A)** Time courses of open circuit potential measurements for the sediment containing oligochaetes and sediment microorganisms. Forty oligochaetes specimens were collected at 4, 8, 11, and 16 h for nuclear magnetic resonance (NMR) analysis. Red line: sample I at a high redox potential before feeding; light blue line: sample II at a low redox potential after feeding; green line: sample III at the mid-redox potential during recovering; and magenta line: sample IV at a high redox potential after recovery. **(B)** Changes in the relative intensity of the NMR signals for succinate (squares) and fumarate (triangles).

To investigate the role of the digestive activity of oligochaetes in the observed homeostatic changes in sediment redox potential, we analyzed the metabolites of *T.* cf. *briani* using NMR developed for the analysis of complex environmental samples (Simpson et al., 2011; Komatsu et al., 2015; Kikuchi and Yamada, 2017). Forty *T.* cf. *briani* specimens were collected from EC reactors at four different potential regions: high potential before feeding (region I), low potential after 10-mg feeding (region II), mid-potential during recovery (region III), and high potential after recovery (region IV; Figure 4A). The collected specimens were gently washed with artificial seawater to remove excess material and transferred to 2-ml safe lock tubes for NMR analysis. Twenty metabolites were identified by ^1^H-^13^C-Heteronuclear Single Quantum Coherece (HSQC) and ^1^H-^13^C-HSQC-Total Correlation Spectroscopy (TOCSY) NMR (Chikayama et al., 2010; Kikuchi et al., 2016; Supplementary Figure 5). In the 2D*-J*res spectra, succinate and fumarate, which are metabolites of tricarboxylic acid cycle (TCA) cycle, showed clear potential-dependent accumulation (Figure 4B). Namely, the amount of succinate increased with decreasing potential in regions I to II; however, the levels returned to the original one as the potential increased in regions III and IV. A positive correlation between fumarate levels and sediment potential was observed. Namely, the amount of fumarate decreased with decreasing potential, but subsequently increased with an increase in the sediment potential.

Fumarate respiration has been found in several benthic organisms, such as mussels, and is thought to sustain cellular functions under hypoxic conditions (Holwerda and De Zwaan, 1980; Van Hellemond and Tielens, 1994). In this mechanism, fumarate serves as an electron acceptor to form succinate, generating a proton motive force for adenosine triphosphate (ATP) synthesis. In the sediment of our model benthic ecosystem, the accumulation of succinate was coupled with a decrease in fumarate under low potential conditions, suggesting that *T.* cf. *briani* gains energy by digesting organic matter *via* fumarate respiration under hypoxic conditions. However, when low-potential conditions last for 15 days or more, most of the *T.* cf. *briani* were found to die.

### Electrochemical regulation of metabolism and movement

**Figure 5:**
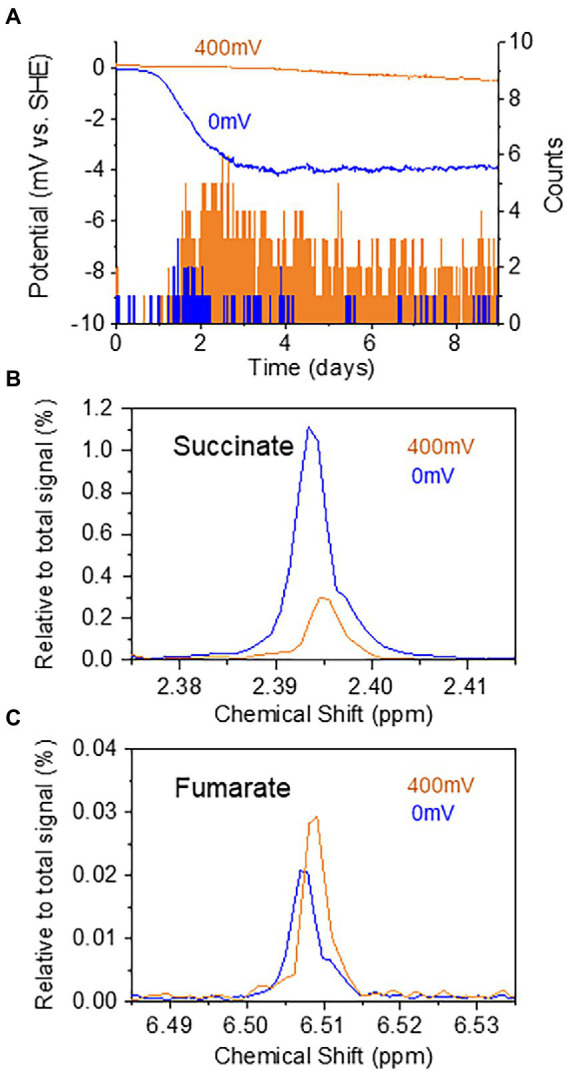
**(A)** Time courses of current generation from the sediment containing oligochaetes and sediment microorganisms at 0 and 400 mV. The number of oligochaetes with tails protruding from the sediment into the sea water at 0 and 400 mV is indicated by orange and blue bars, respectively. **(B,C)** Potential-dependent changes in the relative intensity of the NMR signals for succinate **(B)** and fumarate **(C)**.

The results presented above have demonstrated the effectiveness of real-time potential monitoring to assess the homeostatic function of benthic ecosystems. As a final experiment, the benthic ecosystem containing *T.* cf. *briani* and sediment microorganisms was maintained at electrochemical potentials of 0 or 400 mV to examine if the movement and metabolism of *T.* cf. *briani* could be artificially regulated. Upon applying the potential to the sediment-electrode system, a cathodic current of approximately −3.8 and − 0.5 μA was generated at 0 and 400 mV, respectively (Figure 5A). In addition, the movement of *T.* cf. *briani* clearly differed between 0 and 400 mV, with several oligochaetes extending their tails into the seawater at 400 mV (orange: six of 10 individuals), whereas their entire bodies were buried in the sediment at 0 mV. This potential-dependent regulation of movement is consistent with the change in movement that was observed upon feeding (Figure 3B). After 16 days of electrochemical incubation at 0 and 400 mV, 40 specimens of *T.* cf. *briani* were collected from the sediment and NMR analysis was conducted to examine changes in fumarate and succinate levels at the two sediment potentials. A higher amount of succinate was detected in *T.* cf. *briani* in the sediment with an applied potential of 0 mV relative to those organisms from sediment subjected to 400 mV, whereas fumarate showed the opposite trend (Figures 5B,C). This finding is consistent with the potential-dependent change in metabolite levels that was induced by feeding (Figure 4B), providing strong evidence that the redox homeostasis that observed upon introducing organic matter into the sediment originates from the redox-dependent activity of *T.* cf. *briani*.

Based on the real-time monitoring of redox potential changes upon nutrient addition and the observed electrochemical regulation of metabolism and movement of *T.* cf. *briani*, we propose the following mechanism of redox homeostasis in the model benthic ecosystem. The degradation of organic compounds by *T.* cf. *briani* results in reversible changes in metabolic states and body positions, together leading to changes in the sediment potential. Under high-potential conditions, *T.* cf. *briani* extend its tail into the oxygenated seawater and gains energy by digesting organic compounds *via* aerobic respiration. The introduction of organic compounds into the ecosystem leads to a large drop in the redox potential of the sediment, likely due to the enrichment of anaerobic microorganisms, such as sulfate-reducing bacteria, as inferred from the observed formation of sulfide ions and FeS. In contrast, low-potential conditions were associated with *T.* cf. *briani* embedded in the sediment, a switch from aerobic respiration to the use of fumarate as an electron acceptor. The digestion of organic compounds *via* fumarate respiration leads to an increase in the sediment potential, and the extension of its tail into the seawater-sediment interface facilitates aerobic respiration by *T.* cf. *briani*. This redox-dependent reversible switching in metabolism and movement showcases the high versatility of oligochaetes to adapt to the benthic environment, which can experience dynamic changes in the redox potential between −300 and 500 mV, and is likely a significant factor for maintaining ecosystem-level homeostasis in marine sediments.

## Summary

In the present study, we demonstrated that the real-time monitoring of redox homeostasis is a viable method for assessing the resilience of benthic ecosystems, which was reinforced by the cooperative relationships between oligochaetes and sediment microorganisms. In particular, the redox-dependent reversible changes in metabolism and movement of oligochaetes was found to play a crucial role in the homeostatic response of the system to nutrient influx. We also demonstrated that it may be possible to artificially manipulate the metabolism and movement of benthic animals through electrochemical control of sediment potential. Recent advances in electromicrobiology have demonstrated the application of electrochemical techniques to the monitoring and regulation of bacterial activity, particularly for metal reducing bacteria with the ability of extracellular electron transfer, such as members of the genus *Shewanella* and *Geobacter* (Nealson and Saffarini, 1994; Bond and Lovley, 2003). The electrochemical control of microbial flora has also been reported in mixed-culture systems, in which specific microbial species were enriched by regulating the electrode potential (Torres et al., 2009). To our knowledge, the present study is the first to demonstrate the electrochemical monitoring and physiologic regulation of benthic animals. Future research on the redox-dependent activity of benthic animals, including polychaetes and amphipods, will allow electromicrobiological techniques to be applied to benthic ecosystems and may lead to the development of novel technologies and sustainable aquaculture practices.

## Data availability statement

The raw data supporting the conclusions of this article will be made available by the authors, without undue reservation.

## Author contributions

NS, KI, and RN conceived and designed the experiments. NS, MI, AU, and KS performed the experiments. NS, MI, KS, AL, JK, KI, and RN analyzed the data. NS and RN wrote the manuscript. All authors contributed to the article and approved the submitted version.

## Funding

This work was supported by a grant from commissioned project study on “Technological developments for characterization of harmful plankton in the seawater (Grant Number JP005317),” Ministry of Agriculture, Forestry and Fisheries, Japan.

## Conflict of interest

The authors declare that the research was conducted in the absence of any commercial or financial relationships that could be construed as a potential conflict of interest.

## Publisher’s note

All claims expressed in this article are solely those of the authors and do not necessarily represent those of their affiliated organizations, or those of the publisher, the editors and the reviewers. Any product that may be evaluated in this article, or claim that may be made by its manufacturer, is not guaranteed or endorsed by the publisher.
