# Supplementary material for: Tracing and regulating redox homeostasis of model benthic ecosystems for sustainable aquaculture in coastal environments
Source: Front Microbiol. 2022 Aug 10;13:907703. doi: 10.3389/fmicb.2022.907703 (PMC9399343; doi:10.3389/fmicb.2022.907703)
Supplement: Supplementary file 1 [file Data_Sheet_1.pdf]

Supplementary Material

1 Supplementary Figures

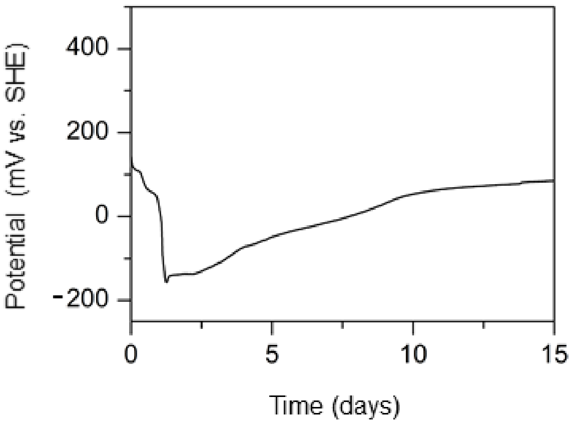

Supplementary Figures 1. Time course of open circuit potential measurements for the autoclaved sediment lacking oligochaetes (i.e., neither sediment microbes nor oligochaetes present in the sediment).

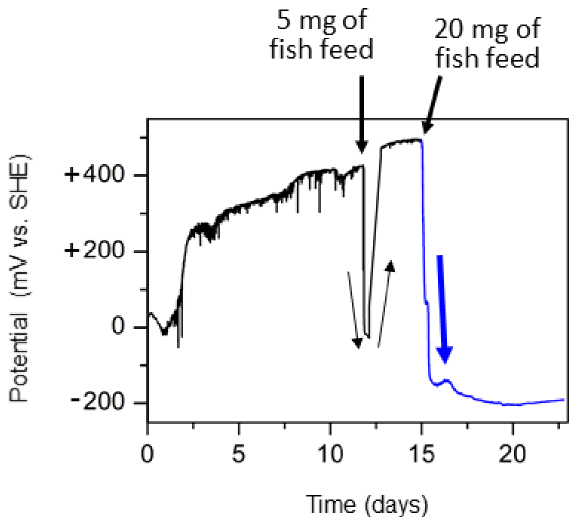

Supplementary Figures 2. Time course of open circuit potential measurements for the sediment containing both oligochaetes and sediment microorganisms. The arrow indicates the time at which 5 and 20 mg of fish diet, respectively, was added to the electrochemical reactor.

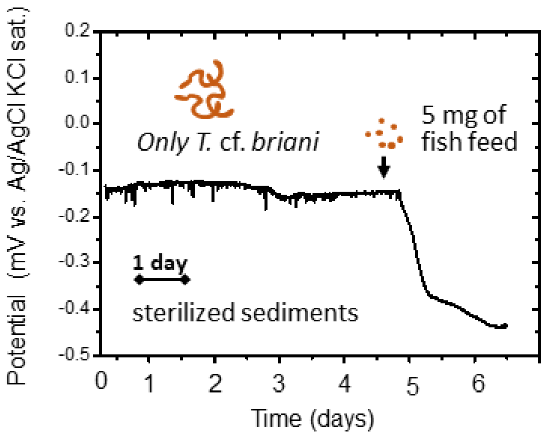

Supplementary Figures 3. Time course of open circuit potential measurements for the sediment containing only oligochaetes. The arrow indicates the time at which 5 mg of fish diet was added to the electrochemical reactor.

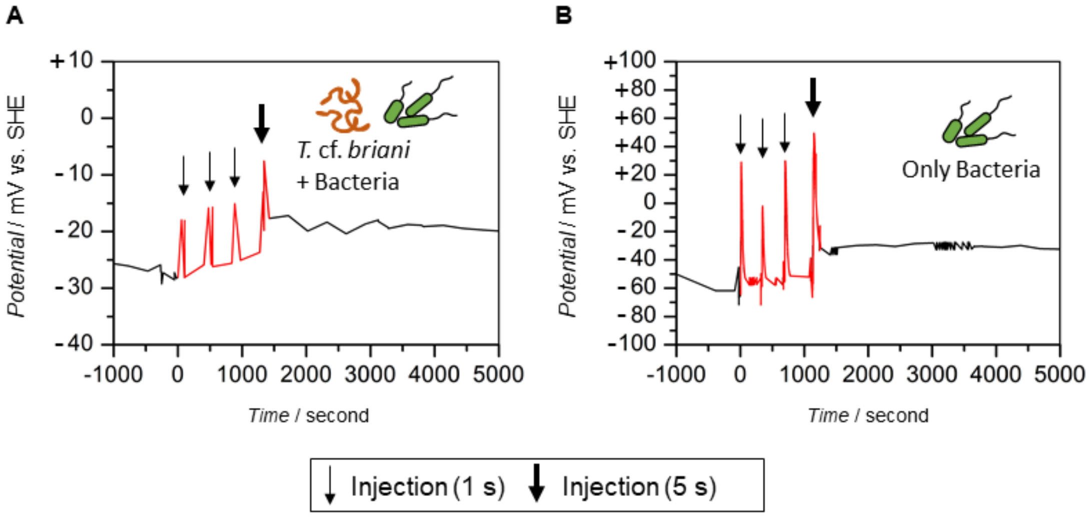

Supplementary Figures 4. The effect of mechanical agitation of the seawater-sediment interface in the EC reactor on open circuit potential. Time courses of open circuit potential measurements for (A) sediment containing both oligochaetes and sediment microorganisms and (B) sediment containing only sediment microorganisms. The agitation was conducted using a microsyringe at an injection rate of 0.2 ml/s. The arrow indicates the time at the injection was conducted for 1 or 5 s.

NMR assignment

A

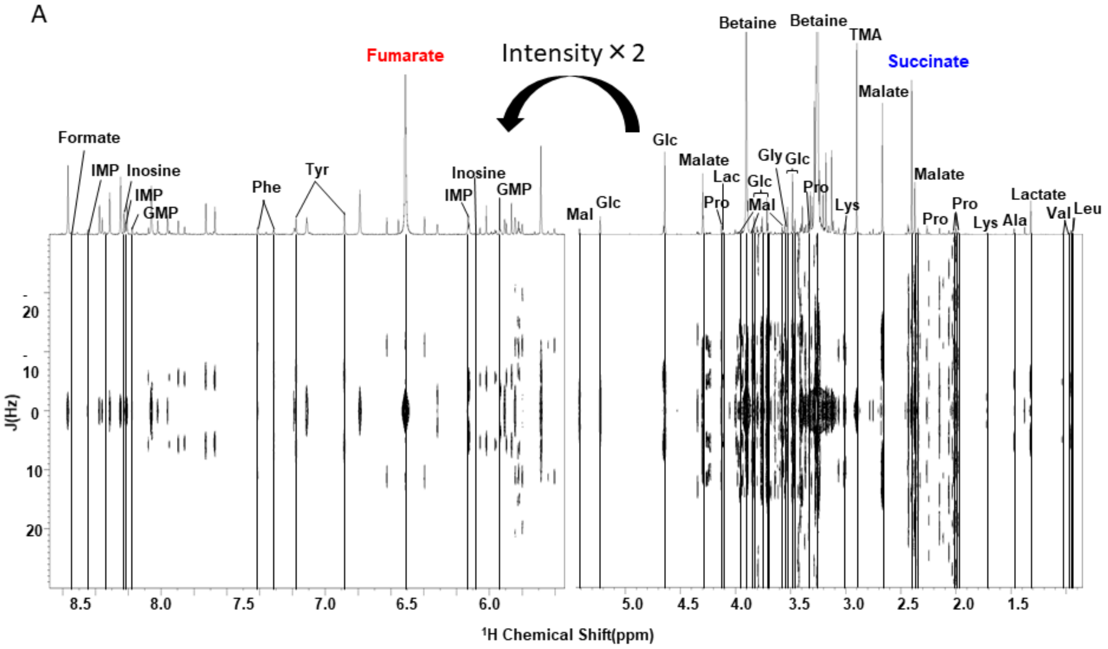

B

Compound name	Chemical shift(ppm)		
Alanine	1.47(d)	3.77(q)	
Gly	3.55(s)		
Tyr	6.89(d)	6.89(d)	7.18(d)
	7.18(d)		
Proline	1.98(m)	2(m)	2.02(m)
	2.06(m)	2.34(m)	3.33(m)
	3.37(m)	3.41(m)	4.13(dd)
Lys	1.43(m)	1.43(m)	1.89(m)
	3.01(t)	3.74(t)	
Leucine	0.96(d)	1.71(m)	3.72(m)
Phe	7.31(d)	7.36(t)	7.41(t)
Valine	0.98(d)	1.03(d)	
Formate	8.45(s)		
Fumarate	6.51(s)		
Lactate	1.32(d)	4.1(q)	
Malate	2.37(dd)	2.67(dd)	4.3(dd)
Succinate	2.4(s)		
Betaine	3.26(s)	3.9(s)	
Trimethylamine	2.9(s)		
GMP	5.93(d)	8.18(s)	
IMP	6.13(d)	8.22(s)	8.55(s)
Inosine	6.09(d)	8.23(s)	8.34(s)
Glucose	3.24(d)	3.39(t)	3.46(m)
	3.48(t)	3.52(dd)	3.7(t)
	3.71(m)	3.76(m)	3.83(m)
	3.89(m)	4.64(d)	5.22(d)
Maltose	3.56(m)	3.57(m)	3.58(m)
	3.68(m)	3.69(m)	3.8(m)
	3.85(m)	3.9(m)	3.96(m)
	4.65(d)	5.41(d)	

Supplementary Figures 5. Assignment of NMR signals in Oligochaetes extracts. (A) 1H-13C-HSQC and 1H-13C-HSQC-TOCSY NMR were applied to samples extracted from Oligochaetes prior to use in the experiment. Signals of 20 metabolites shown in the figure could be identified. (B) A list of identified metabolites and their chemical shifts.

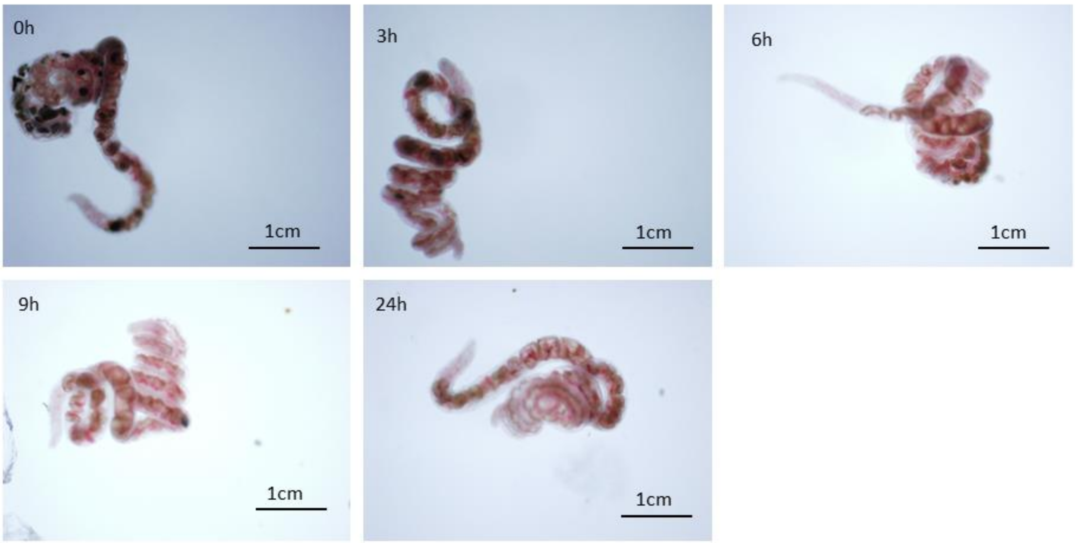

Supplementary Figures 6. The light microscope images of the specimen of the marine oligochaete *Thalassodrilides* cf. *briani* shows the processes that *T*. cf. *briani* excretes sediments from the body.

2 Supplementary Video Caption

Supplementary Video 1. A video showing *Thalassodrilides* cf. *briani* digging into the sediment.
